# Impact of lifestyle Intervention on branched‐chain amino acid catabolism and insulin sensitivity in adolescents with obesity

**DOI:** 10.1002/edm2.250

**Published:** 2021-04-01

**Authors:** Catherine Jachthuber Trub, Metin Balikcioglu, Michael Freemark, James Bain, Michael Muehlbauer, Olga Ilkayeva, Phillip J. White, Sarah Armstrong, Truls Østbye, Steven Grambow, Pinar Gumus Balikcioglu

**Affiliations:** ^1^ Emory University Medical Center Atlanta GA USA; ^2^ Advanced Analytics Division SAS Institute Inc Cary NC USA; ^3^ Division of Pediatric Endocrinology and Diabetes and the Duke Molecular Physiology Institute Duke University Medical Center Durham NC USA; ^4^ Duke Molecular Physiology Institute Duke Molecular Physiology Institute Duke University Medical Center Durham NC USA; ^5^ Division of General Pediatrics Duke University Medical Center Durham NC USA; ^6^ Department of Family Medicine and Community Health Duke University Medical Center Durham NC USA; ^7^ Department of Population Health Sciences Duke University Medical Center Durham NC USA; ^8^ Duke Clinical Research Institute Duke University Medical Center Durham NC USA; ^9^ Department of Biostatistics and Bioinformatics Duke University Medical Center Durham NC USA

**Keywords:** BCAA, childhood obesity, insulin resistance

## Abstract

Insulin resistance in adolescents with obesity associates with a sex‐dependent metabolic ‘signature’ comprising branched‐chain amino acids (BCAAs), glutamate and C3/C5 acylcarnitines (C3/C5), implicating altered flux through BCAA catabolic pathways. Here, we investigated the effects of lifestyle intervention on BCAA catabolism and insulin sensitivity. We hypothesized (1) weight reduction and improved insulin sensitivity associate with enhanced BCAA catabolism; (2) baseline BCAAs and their metabolic by‐products predict changes in weight and insulin sensitivity during lifestyle intervention.

**Methods:**

A 33 adolescents with obesity were studied before and after 6 months of lifestyle intervention. Principal component analysis and multiple linear regression models were used to correlate changes in metabolic factors with changes in weight and insulin sensitivity assessed by HOMA‐IR, adiponectin and ratio of triglyceride (TG) to HDL. Baseline metabolic factors were used as explanatory variables in prediction models.

**Results:**

Weight reduction was associated with reductions in BCAA, glutamate, and C3/C5 (*p* = .002) and increases in urea cycle AA (*p* = .029), suggesting an increase in BCAA catabolism. Increases in urea cycle AA during weight reduction were associated with increases in adiponectin, a marker of insulin sensitivity. Markers of insulin resistance (high BCAA, glutamate, and C3/C5 and low urea cycle AA) at baseline predicted increases in metrics of insulin sensitivity (decreased TG/HDL and increased adiponectin) during lifestyle intervention.

**Conclusions:**

Weight reduction in adolescents is associated with increases in BCAA catabolism and improvements in insulin sensitivity. Our study underscores the therapeutic potential of manipulating BCAA catabolism to treat obesity‐associated insulin resistance in adolescents and prevent progression to T2D.

## Graphical abstract

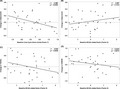

## 1 INTRODUCTION

Obesity and insulin resistance (IR) are the major risk factors for paediatric (and adult) type 2 diabetes (T2D)^1^, ^2^; however, only half of youth with obesity are insulin resistant and even fewer (2%–8%) progress to T2D.^3^, ^4^ Thus, obesity is neither sufficient nor sensitive for predicting who will become insulin resistant and metabolically unhealthy,^5^ and who will develop T2D. Additional factors, such as disproportionate body fat distribution and/or increased liver fat content and visceral fat mass, are essential for progression to overt glucose intolerance.^6^

Using state‐of‐the‐art targeted metabolomic profiling, principal components analysis (PCA) and regression analysis, we previously showed that IR in adolescents with obesity is associated with a sex‐dependent metabolic ‘signature’ comprising the branched‐chain amino acids (BCAAs), glutamate and C3/C5 acylcarnitines, implicating an altered flux through the BCAA catabolic pathway.^7^ We called this metabolic signature ‘BCAA‐related factor’, which in our PCA was Factor 2. Elevated levels of BCAA and the other components of PCA Factor 2 associate with insulin resistance in obese adults as well as adolescents.^7^, ^8^, ^9^, ^10^ As in adults, it is not clear if high BCAA levels in adolescents are a cause or a consequence of obesity and IR or both,^11^, ^12^, ^13^ or if levels can be modified by dietary/exercise intervention.^13^, ^14^, ^15^, ^16^ However, recent work in rat and mouse models of obesity and IR demonstrates that enhanced catabolism of BCAA mediated by pharmacologic activation of the branched‐chain α‐keto acid dehydrogenase (BCKDH) improves insulin sensitivity while lowering circulating BCAAs and branch chain ketoacids (BCKAs).^17^, ^18^

Building on our novel findings, we investigated here the relationship between BCAA catabolism and insulin sensitivity during lifestyle intervention. We hypothesized that: (1) weight reduction and improved insulin sensitivity during intervention are associated with enhanced BCAA catabolism, evident by decreases in BCAAs and their metabolic by‐products (PCA Factor 2); and (2) baseline BCAAs and their metabolic by‐products predict subsequent changes in weight and insulin sensitivity. To test these hypotheses, we used targeted metabolomic profiling, PCA and multiple linear regression models to assess the correlations between changes in metabolic factors and changes in weight and insulin sensitivity as assessed by homeostasis model assessment index of insulin resistance (HOMA‐IR), adiponectin and the ratio of triglyceride (TG) to HDL. In prediction models, metabolic factors at baseline were used as explanatory variables. We also stratified the data by sex to see whether there are differences that are not detected when data from both sexes are pooled.^19^, ^20^

## 2 RESEARCH DESIGN AND METHODS

### 2.1 The Duke Children's Healthy Lifestyles program

The Duke Children's Healthy Lifestyles programme (HLP) provides comprehensive clinical care for children and adolescents with overweight and obesity and represents the current standard of clinical care for paediatric obesity treatment. Treatment in the HLP uses motivational interviewing to modify dietary and activity behaviours in order to reduce the severity of overweight or obesity and obesity‐related comorbidities.^21^ At the initial visit to the HLP (1 h), the medical provider meets with the family to review a comprehensive lifestyle, birth, medical, family and social history; conduct a physical examination focussed on obesity‐related conditions; and screen for psychological and disordered eating concerns. The provider discusses the meaning of the patient's BMI (weight‐for‐length if the patient is <2 years old) as a function of age and sex and assesses the patient's risk for disease in the context of the described behaviours, the laboratory studies and the family history. In addition, the medical provider provides medical management of obesity‐associated comorbidities, as necessary. Patients also meet with a registered dietician and paediatric physical therapist to complete a nutrition and fitness assessment, and recommendations are tailored to the individual family needs. Nutritional guidance follows standard recommendations as provided by the American Academy of Pediatrics.^22^

Families are encouraged to follow‐up with the Healthy Lifestyles team monthly for 1 year. The medical provider provides ongoing management of obesity‐associated comorbidities and lifestyle modification (30 min), and the patient meets with a registered dietician for medical nutritional therapy (30 min). The focus of these visits is patient‐centred goal setting around evidence‐based lifestyle habits known to influence obesity such as reducing sugar‐sweetened beverages and screen time, and increasing fruit and vegetable consumption and physical activity. Families in Healthy Lifestyles are also invited to activity sessions and cooking classes at a community recreation centre. These sessions are adapted for children with obesity and aim to provide hands‐on experience preparing healthy foods and at least 45 min of moderate to vigorous physical activity. Sessions are offered 6 days per week, and families are encouraged to attend at least 2 sessions per week.

### 2.2 Patient cohort

Participants were identified prior to enrolment in Duke Children's Healthy Lifestyles Program and followed prospectively for 6 months. Inclusion criteria stipulated that the participant was new to the Healthy Lifestyles Program, ≥12 to 18 years of age, and overweight or obese (defined as BMI≥85^th^ percentile for sex and age according to CDC growth charts), and that the participant and at least one parent/guardian were able to speak/read English fluently enough to understand and complete questionnaires and intake forms. Exclusion criteria included a diagnosis of T2D and/or use of weight‐reducing agents, systemic corticosteroids, atypical antipsychotics, oral contraceptives or medroxyprogesterone within the past 6 months. Participation was terminated if the subject did not provide fasting blood samples within 2 weeks of his/her first clinic visit. Informed consent was obtained from at least one parent/guardian for each participant <18‐year‐old and from participants ≥18‐year‐old. The Duke University IRB approved the research protocol. 82 overweight and obese adolescents provided baseline data. 33 participants (16 males and 17 females) completed the study and provided fasting plasma samples at baseline and 6 months.

### 2.3 Blood samples

Baseline and 6‐month follow‐up blood samples were obtained after an 8‐ to 12‐h overnight fast. Plasma was stored at −80°C until analysed.

### 2.4 Anthropometric measurements

Body weight and height were measured by standard methods. Blood pressure was measured twice; the average was used in statistical analyses. Age, sex and height‐specific normal values for children are available at https://www.nhlbi.nih.gov/files/docs/bp_child_pocket.pdf. Body fat percentage was estimated using a Tanita BC‐148 segmental body composition analyzer. BMI, BMI percentile (BMI %), BMI z‐score and the per cent BMI exceeding the 95^th^ %ile were calculated using the SAS programme (https://www.cdc.gov/nccdphp/dnpao/growthcharts/resources/sas.htm). Our cohort consisted of subjects with extreme BMI values, in whom BMI percentile and BMI z‐score provide unreliable estimates of the degree of overweight and the response to intervention.^23^, ^24^ Consequently, we used ‘BMI per cent exceeding the 95^th^ percentile’ for age and sex to track the weight changes over time.

### 2.5 Hormone analysis

Hormones were measured using a Meso Scale Discovery Quick Plex electro chemiluminescent imager with assay kits from Meso Scale Discovery (Rockville, MD) including insulin (range 69–50,000 pg/ml), leptin (range 137–100,000 pg/ml) and total adiponectin (range 0.064–1000 ng/ml, and samples diluted 1:961). Duplicate measurements had coefficients of variations <10%. HOMA‐IR was calculated as fasting insulin (μU/ml) multiplied by fasting glucose (mg/dl) divided by 405.^25^

### 2.6 Conventional metabolite analysis

Conventional metabolites, including plasma glucose, total cholesterol, HDL, LDL, TGs, lactate, uric acid and high sensitivity C‐reactive protein (hsCRP), were measured with a Beckman Coulter DxC 600 Clinical Analyzer using reagents from Beckman (Brea, CA), as well as total nonesterified fatty acids (NEFA), total ketone bodies and 3‐hydroxybutyrate using reagents from Wako (Mountain View, CA). Coefficients of variation were <5%.

### 2.7 Plasma acylcarnitines and amino acids

A 45 Acylcarnitines (0.01–40 µmol/L, <15%) and 15 amino acids (5–1000 µmol/L, <15%) were analysed by tandem mass spectrometry (MS/MS) using a Waters TQD instrument. Amino acids and acylcarnitines were analysed by flow injection electrospray ionization tandem mass spectrometry and quantified by isotope or pseudo‐isotope dilution using methods described previously.^26^, ^27^ Briefly, plasma samples were spiked with a cocktail of heavy‐isotope internal standards (Cambridge Isotope Laboratories; CDN Isotopes, Canada) and deproteinated with methanol. The methanol supernatants were dried and esterified with either acidified methanol or butanol for acylcarnitine or amino acid analysis, respectively. Mass spectra for acylcarnitine and amino acid esters were obtained using precursor ion and neutral loss scanning methods, respectively. The data were acquired using a Waters TQ (triple quadrupole) detector equipped with AcquityTM UPLC system and a data system controlled by MassLynx 4.1 operating system (Waters). Ion ratios of analyte to respective internal standard computed from centroided spectra were converted to concentrations using calibrators constructed from authentic aliphatic acylcarnitines and amino acids (Sigma; Larodan, Sweden) and dialysed Foetal Bovine Serum (Sigma).

Assays were run in a 96‐well‐plate format, with a calibration curve and a set of two QC samples at the beginning and end of each plate. Use of two independent QC samples enabled monitoring of intra‐ and interday precision of the assay. Over the course of several recent years, intra‐ and interday CV of acylcarnitine measurements in QC plasma were <15%, and <10% for abundant analytes, such as plasma amino acids and acetylcarnitine.

### 2.8 Statistical analysis

Principal components analysis was used to reduce the large number of correlated metabolites into clusters of fewer components not correlated with each other. The metabolic factors and metabolites comprising these factors are described in our previous study.^7^ Minimum sample size was calculated to detect correlations of 0.5 or greater between the ‘BCAA‐related factor’ (PCA Factor 2) and insulin sensitivity. In linear regression models using 4 explanatory variables with one testing variable, a sample size of 32 provides a correlation of 0.5 with power of 0.8 and *p* < .05. Thus, our sample size of 33 provided adequate statistical power.

Metabolites measured at 6‐month follow‐up were scored using the PCA results obtained in the original study to construct the same factors. These metabolic factors served as explanatory variables. HOMA‐IR, adiponectin and TG /HDL ratio were natural log transformed to approximate normality. Multiple linear regression models were used to analyse the associations between changes in metabolic factors, changes in surrogate measures of IR and changes in weight. All models were adjusted for age, sex and BMI z. Models for change in insulin sensitivity were also adjusted for change in BMI% exceeding the 95^th^ percentile. Likewise, the model for change in BMI% exceeding the 95^th^ percentile was also adjusted for change in HOMA‐IR. Statistically significant changes in factors were determined using stepwise linear regression analysis. To investigate if baseline factors predict subsequent changes in insulin sensitivity in response to lifestyle intervention, metabolic factors at baseline were used as explanatory variables in linear regression models.

Anthropometric values and metabolites related to BCAA catabolism across time were compared using paired *t* tests. Data were stratified by sex for analysing the effects separately for females and males. Unpaired t tests were used to compare anthropometric values and metabolites related to BCAA catabolism among females and males at baseline and at 6‐month follow‐up. Baseline anthropometric values and metabolites among participants with and without follow‐up were compared using unpaired t test to address selection bias (Table S1). For all analyses, *p* < .05 was considered statistically significant; analyses were performed using SAS version 9.4 (SAS Institute Inc.).

## 3 RESULTS

### 3.1 Response to lifestyle intervention: comparisons of anthropometric values and metabolic characteristics at baseline and follow‐up

In response to lifestyle intervention, ‘BMI% exceeding the 95^th^ percentile’ decreased in 18 participants and increased in 15. There were no significant changes in BMI‐related metrics. Mean systolic blood pressure decreased (minus 4.48 mmHg, *p* = .0385). Metabolites and surrogate measures of insulin sensitivity at follow‐up were comparable to those at baseline (Table 1).

**TABLE 1:** Comparisons of anthropometric values, insulin sensitivity measures and metabolites, at baseline and follow‐up

	Baseline Mean (SE), *n* = 33	Follow‐up Mean (SE), *n* = 33	*p*‐value
Anthropometric values			
Age, years	14.20 (0.25)	14.71 (0.25)	**<.0001**
BMI	34.67 (1.17)	34.85 (1.21)	.5108
BMI %	98.31 (0.29)	98.27 (0.24)	.8025
BMI Z‐score	2.27 (0.07)	2.25 (0.07)	.3586
BMI% exceeding the 95^th^ percentile	129.75 (4.14)	128.30 (4.24)	.1674
% BF	41.21 (1.49)	41.88 (1.62)	.7147
Systolic BP, mmHg	120.30 (2.08)	115.82 (1.71)	**.0385**
Diastolic BP, mmHg	64.73 (1.66)	64.65 (1.39)	.9523
Insulin sensitivity measures			
Adiponectin, μg/ml	15.90 (1.28)	16.31 (1.20)	.6026
HOMA‐IR	4.04 (0.62)	5.20 (1.29)	.1310
TG to HDL ratio	1.85 (0.22)	1.89 (0.26)	.7800
Metabolites			
Glutamate/glutamine μM	35.17 (2.06)	34.08 (1.53)	.4529
Valine, μM	118.27 (3.37)	116.76 (3.91)	.7193
Leucine/Isoleucine, μM	84.31 (2.28)	84.39 (2.63)	.9731
BCAA, μM	202.58 (5.38)	201.15 (6.33)	.8219
C2 acylcarnitine, μM	3.37 (0.25)	2.84 (0.18)	.0148
C3 acylcarnitine, μM	0.16 (0.01)	0.15 (0.01)	.8472
C5 acylcarnitine, μM	0.07 (0.01)	0.07 (0.01)	.9574

### 3.2 Comparisons of anthropometric values and metabolites by sex

At baseline, males and females were comparable in age, weight and BMI‐related metrics. At baseline and follow‐up, males had higher levels of BCAAs (*p* = .0121) and Glutamate/Glutamine (*p* = .0186), consistent with our previous findings.^7^ C3 (*p* = .0160) and C5 (*p* = .0246) acylcarnitine levels were also higher among males at 6 months.

### 3.3 Principal components analysis

#### 3.3.1 Association models

##### Associations between insulin sensitivity and BCAA‐related factor (PCA Factor 2) at 6‐month follow‐up

Significant metabolic factors identified in our original study served as explanatory variables in the follow‐up regression analysis. Using the 6‐month follow‐up data, BCAA‐related factor (PCA Factor 2) was again significantly associated with HOMA‐IR (*p* = .0050) and the ratio of TG/HDL (*p* = .0344, Table 2). This is consistent with the results of our original study.

**TABLE 2:** Factors associated with HOMA‐IR, adiponectin and TG to HDL ratio at 6‐month follow‐up (Sex, Age and BMI Z‐Score Adjusted)

Full sample	Parameter estimate	*t* value	*p*‐value
HOMA‐IR	*6*‐*month follow*‐*up* (*n* = 33, *R* ^2^ = .587, *p* = .003)		
Female	0.677	2.52	.0186
Age	0.013	0.16	.8763
BMI Z‐Score	0.537	1.64	.1135
Factor 1: FAO by‐products	−0.203	−1.76	.0920
Factor 2: BCAA‐related and uric acid	0.480	3.09	.0050
Factor 11: glucogenic amino acids (PRO ALA)	0.176	1.09	.2857
Factor 12: C5:1, ASX and long‐chain dicarboxyl acylcarnitines	−0.157	−1.09	.2878
Factor 14: Miscellaneous	−0.282	−1.63	.1162
Adiponectin	*6*‐*month follow*‐*up* (*n* = 33, *R* ^2^ = .188, *p* = .313)		
Female	−0.309	−1.68	.1054
Age	−0.024	−0.45	.6536
BMI Z‐Score	−0.130	−0.59	.5571
Factor 2: BCAA‐related and uric acid	−0.175	−1.67	.1056
Factor 4: medium‐chain acylcarnitines	−0.061	−0.64	.5274
TG to HDL Ratio	*6*‐*month follow*‐*up* (*n* = 33, *R* ^2^ = .504, *p* = .001)		
Female	0.233	0.98	.3349
Age	0.067	0.99	.3315
BMI Z‐Score	0.858	3.06	.0050
Factor 2: BCAA‐related and uric acid	0.300	2.23	.0344
Factor 4: medium‐chain acylcarnitines	−0.053	−0.43	.6682

Note

Sex, age and BMI z‐score are included in all linear regression models; however, only statistically significant variables are reported. The *p*‐value included in the parentheses under the parameter estimate column refers to the overall model fit (F statistics).

##### Associations between changes in weight and insulin sensitivity and changes in PCA factors

**FIGURE 1:**
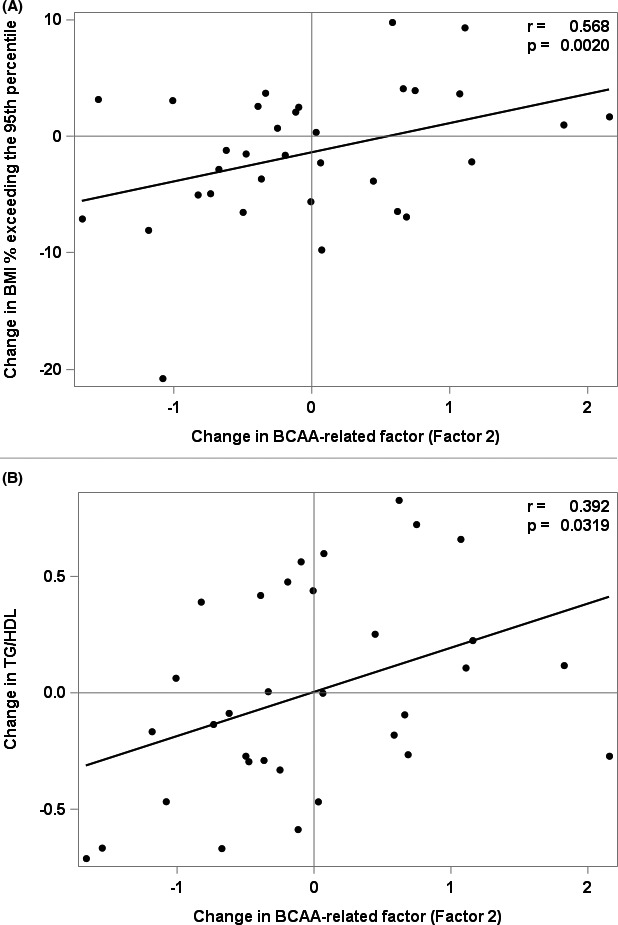
Associations between change in BCAA‐related factor (Factor 2) and change in BMI% exceeding the 95^th^ percentile and change in TG/HDL ratio, r is the partial Pearson correlation coefficient adjusted for Sex, Age, BMI z‐Score and selected significant factors. (A) Associations between change in BMI% exceeding the 95^th^ percentile and change in BCAA‐related factor (Factor 2), (B) Associations between change in TG/HDL and change in BCAA‐related by‐products (Factor 2)

**FIGURE 2:**
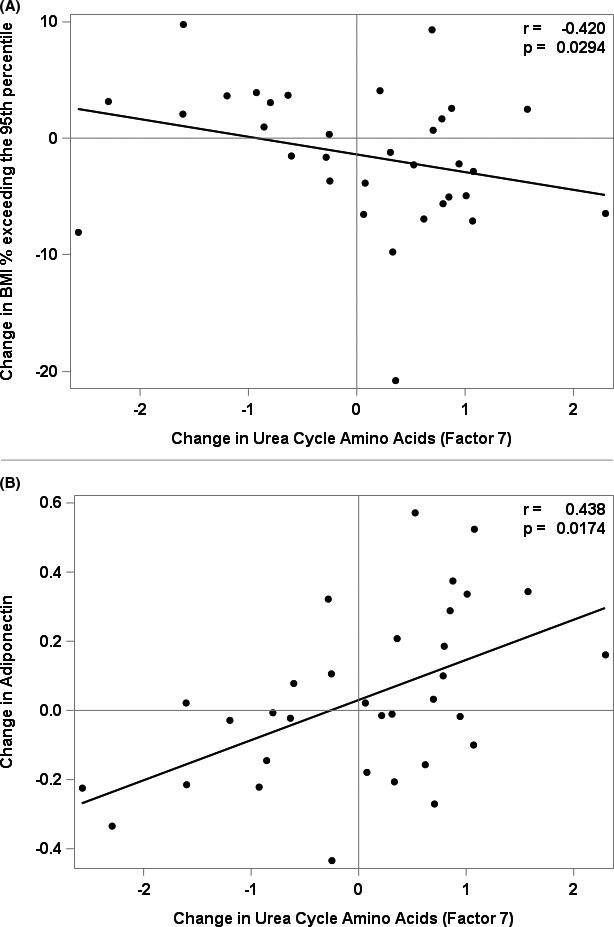
Associations between change in Urea Cycle Amino Acids, and change in BMI% exceeding the 95^th^ percentile and change in Adiponectin, r is the partial Pearson correlation coefficient adjusted for Sex, Age, BMI z‐Score and selected significant factors. (A) Associations between change in BMI% exceeding the 95^th^ percentile and change in Urea Cycle Amino Acids (Factor 7). (B) Associations between change in adiponectin and change in Urea Cycle Amino Acids (Factor 7)

**FIGURE 3:**
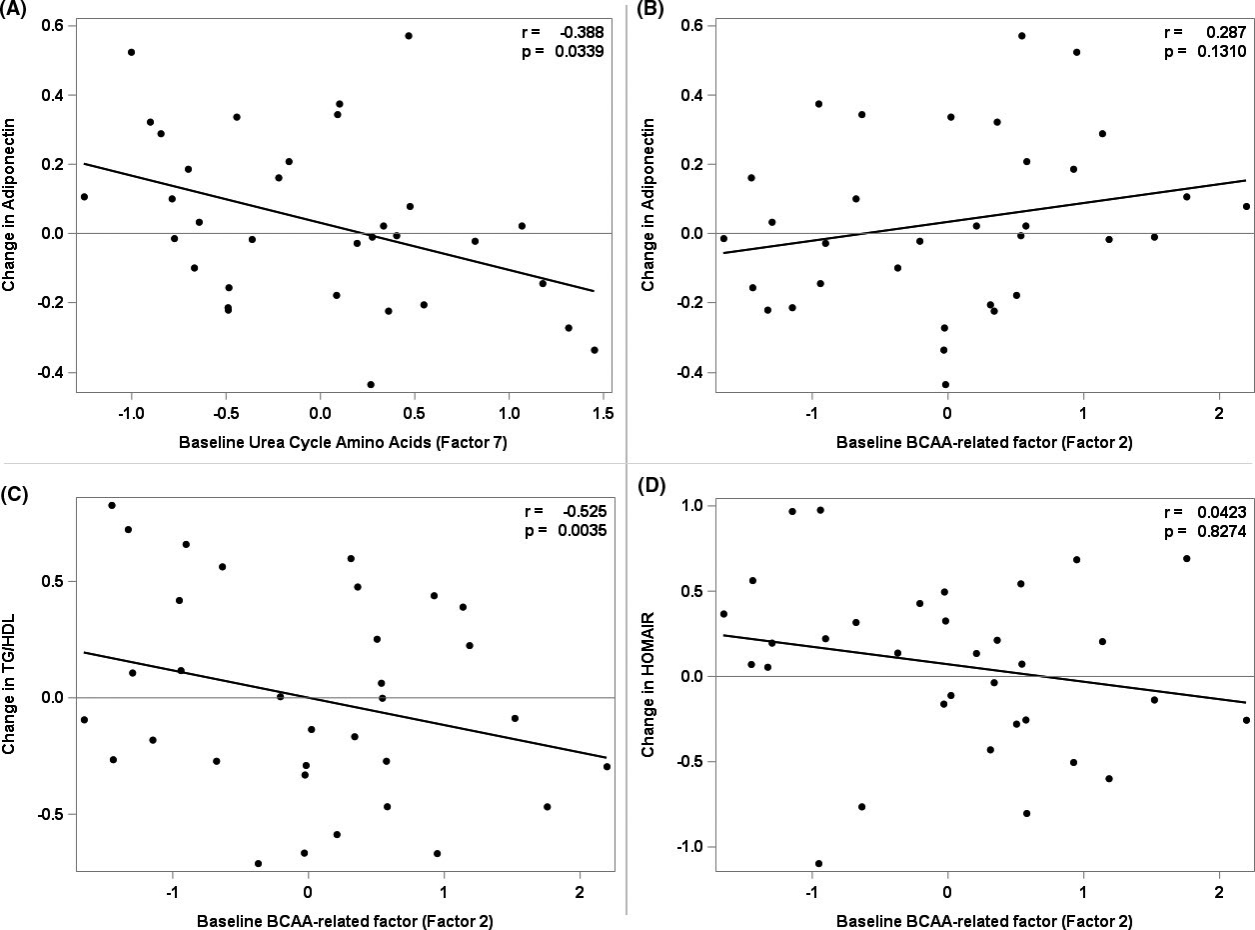
Prediction of surrogate measures of insulin sensitivity with baseline factors, r is the partial Pearson correlation coefficient adjusted for Sex, Age, BMI z‐Score and selected significant factors. (A) Prediction of insulin sensitivity assessed by change in adiponectin with baseline Urea Cycle Amino Acids (Factor 7). (B) Prediction of insulin sensitivity assessed by change in adiponectin with baseline BCAA‐related factor (Factor 2). (C) Prediction of insulin sensitivity assessed by change in TG/HDL with baseline BCAA‐related factor (Factor 2). (D) Prediction of insulin sensitivity assessed by change in HOMA‐IR with baseline BCAA‐related factor (Factor 2)

Figures 1, 2, 3 and 2 and Table 3A summarize the association models selected for change in ‘BMI% exceeding the 95^th^ percentile’, HOMA‐IR, adiponectin and the TG/HDL ratio. Reduction in ‘BMI% exceeding the 95^th^ percentile’ during lifestyle intervention was associated with decreases (*p* = .0020) in BCAAs and their metabolic by‐products (PCA Factor 2) and increases (*p* = .0294) in urea cycle amino acids (PCA Factor 7, Table 3A). These were accompanied by increases in metrics associated with heightened insulin sensitivity: a decrease in BCAAs and their metabolic by‐products (PCA Factor 2) was associated with a decrease in the ratio of TG/HDL (*p* = .0319), while an increase in urea cycle amino acids (PCA Factor 7) was associated with an increase in adiponectin (*p* = .0174). Thus, reductions in ‘BMI% exceeding the 95th percentile’ and increases in metrics of insulin sensitivity were accompanied by decreases in BCAA, glutamate, and the C3/C5 acylcarnitines and increases in urea cycle amino acids. These findings implicate the urea cycle amino acids as novel markers of insulin sensitivity.

**TABLE 3:** Change in factors associated with change in BMI% exceeding the 95^th^ percentile, HOMA‐IR, Adiponectin and TG to HDL Ratio (A) Sex, Age and BMI Z‐Score Adjusted; (B) Adjusted for sex, age, BMI Z‐Score and respective change in ‘BMI% exceeding the 95^th^ percentile’ or change in measures of insulin sensitivity

Full sample	Parameter estimate	t value	*p*‐value
(A)			
Δ BMI% exceeding the 95^th^ percentile	(*n* = 33, *R* ^2^ = .507, *p* = .007)		
Δ Factor 2: BCAA‐related and uric acid	3.416	3.45	.0020
Δ Factor 7: Urea cycle amino acids and HIS	−1.964	−2.31	.0294
Δ Factor 8: Short‐chain acylcarnitines	−3.064	−2.79	.0099
Δ Factor 10: Large neutral amino acids and CRP	2.736	2.13	.0436
Δ HOMA‐IR	(*n* = 33, *R* ^2^ = .297, *p* = .075)		
Δ Factor 3: Medium‐chain acylcarnitines	−0.168	−2.09	.0461
Δ Factor 5: Long‐chain acylcarnitines	−0.332	−2.66	.0129
Δ Adiponectin	(*n* = 33, *R* ^2^ = .497, *p* = .002)		
Δ Factor 6: Medium‐chain acylcarnitines	0.096	2.81	.0091
Δ Factor 7: Urea cycle amino acids and HIS	0.082	2.53	.0174
Δ TG to HDL Ratio	(*n* = 33, *R* ^2^ = .166, *p* = .2607)		
Δ Factor 2: BCAA‐related and uric acid	0.194	2.22	.0319
(B)			
Δ BMI% exceeding the 95^th^ percentile	(*n* = 33, *R* ^2^ = .460, *p* = .008)		
Δ HOMA‐IR	4.329	2.31	.0239
Δ Factor 2: BCAA‐related and uric acid	2.878	2.78	.0099
Δ Factor 8: Short‐chain acylcarnitines	−2.560	−2.87	.0081
Δ HOMA‐IR	(*n* = 33, *R* ^2^ = .495, *p* = .009)		
Δ BMI% exceeding the 95^th^ percentile	0.031	2.38	.0255
Δ Factor 3: Medium‐chain acylcarnitines	−0.164	−2.31	.0294
Δ Factor 5: Long‐chain acylcarnitines	−0.307	−2.62	.0149
Δ Factor 8: Short‐chain acylcarnitines	0.183	2.46	.0211
Δ Adiponectin	(*n* = 33, *R* ^2^ = .497, *p* = .004)		
Δ BMI% exceeding the 95^th^ percentile	0.002	0.26	.7946
Δ Factor 6: Medium‐chain acylcarnitines	0.097	2.77	.0103
Δ Factor 7: Urea cycle amino acids and HIS	0.084	2.46	.0207
Δ TG to HDL Ratio	(*n* = 33, *R* ^2^ = .168, *p* = .388)		
Δ BMI% exceeding the 95^th^ percentile	−0.003	−0.23	.8186
Δ Factor 2: BCAA‐related and uric acid	0.204	2.11	.0447

Note

Sex, age and BMI z‐score are included in all linear regression models; however, only statistically significant variables are reported. The *p*‐value included in the parentheses under the parameter estimate column refers to the overall model fit (F statistics).

Sex, age and BMI z‐score are included in all linear regression models; however, only statistically significant variables are reported. In addition, change in BMI% exceeding the 95t^h^ percentile model adjusted for change in HOMA‐IR and change in measures of insulin sensitivity models adjusted for change in BMI% exceeding the 95^th^ percentile. The *p*‐value included in the parentheses under the parameter estimate column refers to the overall model fit (F statistics).

To determine whether associations between BCAA, urea cycle amino acids and insulin sensitivity are mediated by changes in weight, we adjusted models for change in insulin sensitivity for change in ‘BMI% exceeding the 95^th^ percentile’ (Table 3B). When the model for change in HOMA‐IR was adjusted for change in ‘BMI% exceeding the 95^th^ percentile’, PCA Factor 3 and PCA Factor 5 (medium and long‐chain acylcarnitines, respectively) remained significant factors and PCA Factor 8 (short‐chain acylcarnitines) became significant. These findings support the notion that changes in HOMA‐IR are mediated by, or associated with, changes in acylcarnitines as well as changes in ‘BMI% exceeding the 95^th^ percentile’. When change in adiponectin was adjusted for change in BMI% exceeding the 95^th^ percentile, the changes in urea cycle amino acids and medium‐chain acylcarnitines remained significant. These findings suggest that change in adiponectin is mediated by, or associated with, changes in urea cycle amino acids and acylcarnitines independent of change in ‘BMI% exceeding the 95th percentile’. When change in TG/HDL was adjusted for change in BMI% exceeding the 95^th^ percentile, the changes in BCAA (Factor 2) remained significant (Table 3B). This finding suggests that changes in TG/HDL are mediated by, or associated with, changes in BCAA and catabolic by‐products independent of change in ‘BMI% exceeding the 95th percentile’.

#### 3.3.2 Prediction models

##### Did baseline metabolic factors predict subsequent changes in weight or markers of insulin sensitivity?

Table 4 provides the selected prediction models for the subsequent changes in weight and insulin sensitivity. Baseline BCAAs and their metabolic by‐products (PCA Factor 2) were not significant components in prediction models for subsequent change in ‘BMI% exceeding the 95^th^ percentile’ or IR as assessed by HOMA‐IR or adiponectin (Figure 3B,D). However, subjects with higher baseline BCAAs and their metabolic by‐products (PCA Factor 2) had greater reduction in TG/HDL at follow‐up (Figure 3C, Table 4, *p* = .0035). Low levels of urea cycle amino acids (Factor 7) at baseline predicted an increase in adiponectin during intervention (Figure 3A, Table 4, *p* = .0339). Thus, markers of insulin resistance at baseline (high levels of BCAAs and their metabolic by‐products and low levels of urea cycle amino acids) predicted changes in TG/HDL and adiponectin associated with increased insulin sensitivity.

**TABLE 4:** Baseline factors predicting change in BMI% exceeding the 95^th^ percentile, HOMA‐IR, Adiponectin and TG to HDL Ratio (Sex, Age, and BMI Z‐Score Adjusted)

Full sample	Parameter estimate	t value	*p*‐value
Δ BMI% exceeding the 95^th^ percentile	(*n* = 33, *R* ^2^ = .16, *p* = .2762)		
Factor 17: Miscellaneous	−0.961	−2.12	.0429
Δ HOMA‐IR	(*n* = 33, *R* ^2^ = .27, *p* = .0584)		
Factor 3: Medium‐chain acylcarnitines	0.228	2.83	.0085
Δ Adiponectin	(*n* = 33, *R* ^2^ = .29, *p* = .0422)		
Factor 7: Urea cycle amino acids and HIS	−0.131	−2.23	.0339
Δ TG to HDL Ratio	(*n* = 33, *R* ^2^ = .34, *p* = .0349)		
Factor 2: BCAA‐related and uric acid	−0.278	−3.21	.0035
Factor 3: Medium‐chain acylcarnitines	−0.192	−2.70	.0119

Note

Sex, age and BMI z‐score are included in all linear regression models; however, only statistically significant variables are reported. The *p*‐value included in the parentheses under the parameter estimate column refers to the overall model fit (F statistics).

## 4 DISCUSSION

Studies in adults find that IR and T2D are associated with a metabolic profile consisting of increased plasma concentrations of BCAAs (Val, Leu, and Ile), and the breakdown products of BCAA (C3 and C5 acylcarnitines), and increased glutamate and alanine (Glu and Ala).^28^, ^29^, ^30^ High circulating levels of BCAA in rodents and human adults with obesity are thought to reflect a decrease in BCAA catabolism in liver and adipose tissue^31^, ^32^, ^33^ and/or an increase in BCAA production by an altered microbiome.^13^ Using metabolomic profiling in plasma samples, we showed that IR in adolescents with obesity is associated with a similar metabolic signature comprising BCAAs, glutamate and C3/C5 acylcarnitines implicating an altered flux through the BCAA catabolic pathway. Interestingly, components of the metabolome were associated differentially with IR in teenage boys and girls: fasting BCAAs were higher in boys and correlated most strongly with HOMA‐IR and adiponectin; in contrast, BCAAs in girls correlated most strongly with the TG/HDL ratio.^7^

As in adults, it is not clear if higher BCAAs in adolescents are a cause or a consequence (or both) of obesity and/or IR or if levels can be modified by dietary/exercise intervention.^11^, ^12^, ^13^, ^14^, ^15^, ^16^ In this study, we investigated the changes in the metabolome of youth with obesity 6 months after enrolment into a lifestyle modification programme. We assessed associations between changes in BCAAs and their metabolic by‐products (PCA Factor 2) and subsequent change in weight and IR. We then determined whether levels of BCAAs and their metabolic by‐products (PCA Factor 2) at baseline predicted changes in weight or insulin sensitivity.

Our findings include three novel observations. First, weight reduction (reduction in ‘BMI% exceeding the 95^th^ percentile’) during intervention was associated with decreases in BCAAs, glutamate, and the C3/C5 acylcarnitines (PCA Factor 2) and an increase in urea cycle amino acids (PCA Factor 7). These findings suggest that weight reduction is accompanied by an increase in BCAA catabolism: oxidation of leucine, isoleucine and valine to their respective α‐ketoacids involves conversion of α‐ketoglutarate to glutamate.^34^ Excess glutamate in turn serves as a source of ammonia for generation of citrulline from ornithine in the urea cycle.^35^, ^36^ Thus, it follows that an increase in BCAA catabolism results in increased flux of nitrogen through the urea cycle, explaining why increases in urea cycle amino acids paralleled decreases in BCAAs, glutamate and the C3/C5 acylcarnitines in subjects who lost weight. Likewise, decreases in BCAAs and their metabolic by‐products (PCA Factor 2) and an increase in urea cycle amino acids (Factor 7) were associated with improvements in insulin sensitivity as assessed by TG/DL and adiponectin. Interestingly, a recent investigation found that elevated plasma glutamate levels in adults are associated with increased carotid intima‐media thickness and liver fat content even after adjustment for age, sex, body fat mass and visceral fat mass.^37^ Thus, the decrease of plasma glutamate levels in association with weight reduction may reduce long‐term risks of cardiovascular disease as well as glucose intolerance.

Second, baseline BCAAs and their metabolic by‐products (PCA Factor 2) did not predict subsequent changes in ‘BMI% exceeding the 95^th^ percentile’, HOMA‐IR or adiponectin following lifestyle intervention. However, those with higher baseline BCAAs and their metabolic by‐products had greater reduction in TG/HDL, while low baseline urea cycle amino acids (PCA Factor 7) predicted an increase in adiponectin. Thus, markers associated with insulin resistance (high BCAA, glutamate and C3/C5 and low urea cycle AA) at baseline predicted increases in metrics of insulin sensitivity (decreased TG/HDL and increased adiponectin) during lifestyle intervention.

Finally, sex differences in the BCAA metabolome at baseline persisted during intervention: fasting levels of BCAAs and glutamate were higher in obese teenage boys than obese girls of similar age, and BMI% exceeding the 95^th^ percentile at baseline and at 6‐month follow‐up; C3 and C5 acylcarnitines at 6‐month were also higher in obese teenage boys. Sex differences in BCAA, glutamate and C3/C5 levels in obese teens are currently unexplained but could in theory reflect sex differences in BCAA production by the microbiome and/or catabolism by liver or adipose tissue.^13^, ^17^, ^31^, ^32^, ^33^ The roles of sex steroids in BCAA production or catabolism are poorly understood but could in theory be mediated by effects on fat distribution and liver fat deposition: testosterone promotes visceral fat deposition and hepatic fat accumulation while oestrogen increases subcutaneous fat storage and limits hepatic steatosis.^38^, ^39^, ^40^

As in our original study,^7^ we used HOMA‐IR, adiponectin and the TG/HDL ratio as surrogate measures of IR. Adiponectin regulates hepatic insulin sensitivity, fasting blood glucose levels and fatty acid breakdown; higher levels of adiponectin are associated with lower fasting glucose, higher rates of fatty acid oxidation and increased insulin sensitivity.^41^ The TG/HDL ratio reflects the balance between TG intake (in the form of dietary chylomicrons), TG clearance by peripheral tissues, and TG synthesis and export from the liver.^42^ Transfer of triglyceride from VLDL to HDL particles increases HDL clearance and thereby reduces plasma HDL. HOMA‐IR is a measure of hepatic insulin sensitivity as it reflects fasting insulin and glucose levels.^25^ Thus, the surrogate measures of IR reflect distinct, but overlapping, components of insulin sensitivity regulated at the level of the liver, adipose tissue and skeletal muscle. Given the differential regulation of BCAA catabolism in these tissues in obese states,^31^, ^32^, ^33^ it may not be surprising that correlations between BCAA‐related factor (PCA Factor 2) and urea cycle amino acids (PCA Factor 7) and HOMA‐IR, adiponectin and TG/HDL varied in response to lifestyle intervention.

We do not yet know why those with highest baseline BCAAs and lowest urea cycle amino acids had the greatest increases in insulin sensitivity, as assessed by TG/HDL and adiponectin. We speculate that lifestyle intervention may have had its greatest impact in the most insulin‐resistant participants, who had the lowest rates of BCAA catabolism at baseline with highest levels of BCAAs and lowest levels of urea cycle amino acids.

That changes in BCAA in our study correlated with changes in insulin sensitivity is not surprising given that genetic and acquired variations in BCAA catabolism in adults are associated with insulin resistance and T2D.^11^ The rate‐limiting step of BCAA catabolism is the oxidative decarboxylation of BCKAs to form CoA esters, a reaction catalysed by BCKA dehydrogenase (BCKD) complex.^17^ In a meta‐analysis of 16,596 individuals, a strong association was found between BCAA levels and a SNP near the PPM1 K gene, which encodes the phosphatase that dephosphorylates and activates the BCKDH complex. In subsequent analysis of 47,877 T2D cases and 267,694 controls, a genetically predicted change of one standard deviation in Ile, Leu and Val levels was associated with odds ratios of 1.44, 1.85 and 1.54 for T2D, respectively.^11^

We found that higher baseline BCAAs and their metabolic by‐products (BCAA‐related factor, Factor 2) were associated with, but did not predict, weight reduction as assessed by reduction in BMI% exceeding the 95^th^ percentile; BCAA levels declined in parallel with BMI reduction and increase in insulin sensitivity. Recent studies assessing the value of BCAAs in predicting response to lifestyle intervention in children reach divergent conclusions.^43^ A prospective study of 186 Korean boys identified BCAAs as predictors of future HOMA‐IR and the metabolic syndrome at 2‐year follow‐up.^44^ In contrast, a smaller study of 80 youths found that BCAAs were negatively associated with HOMA‐IR at baseline but not at follow‐up. Other investigators found that increases in tyrosine preceded changes in BCAAs in obese children.^45^ Conversely, Moran‐Ramos et al. identified a metabolic signature comprised of isoleucine, leucine and tyrosine, along with valine, proline, arginine and phenylalanine, that was associated with obesity at baseline and was an independent risk factor of future hypertriglyceridaemia at 2‐year follow‐up.^46^ Consistent with our findings, in a cohort of 396 Finnish girls, leucine and isoleucine levels in childhood were associated with and predicted in early adulthood the ratio of TG/HDL but not HOMA‐IR or fat mass.^47^ Wahl and colleagues found that lower serum concentrations of phosphatidylcholines and smaller waist circumference predicted weight loss.^48^

Our study has several limitations. The sample size was small, in part because high attrition rates are common in studies of adolescents with obesity and IR.^21^, ^49^, ^50^, ^51^ Yet our sample size provided adequate statistical power for our primary outcome, and subjects completing the study were statistically comparable to those who did not complete the study in diverse metrics including age, BMI, BMI %, BMI z, BMI% exceeding the 95th percentile, % body fat, systolic and diastolic blood pressures, conventional metabolites, plasma acylcarnitines and amino acids (Table S1). Female participants were not studied at standard phases of the menstrual cycle. We used surrogate measures of IR; additional methods, including insulin and glucose clamps and iv and oral glucose tolerance tests, might have provided useful information.

Nevertheless, our findings provide novel insights into the effects of lifestyle intervention on branched‐chain amino acid catabolism and insulin sensitivity in adolescents with obesity. BCAA levels in adolescents with obesity are sex‐dependent, and reduction in ‘BMI% exceeding the 95^th^ percentile’ during lifestyle intervention is associated modestly with increases in BCAA catabolism: BCAA levels decline and the urea cycle amino acids rise in parallel with reduction in ‘BMI% above the 95^th^ percentile’ and an increase in insulin sensitivity. Assessment of BCAA catabolism may provide a useful metric for assessing the response to intervention in adolescents with obesity, and modulation of BCAA catabolism might provide new approaches for treating IR and preventing progression to T2D.

## CONFLICT OF INTEREST

CJT, MB, JB, MM, OI, SA, TØ and PGB have no conflicts of interest to declare. Dr Grambow reports receiving consulting fees from Gilead Sciences for serving on multiple data monitoring committees. MF is a co‐investigator on a grant from the American Heart Association that deals with the pathogenesis and treatment of childhood obesity. MF is also the local PI on a Rhythm‐sponsored study of identification and treatment of children and adults with monogenic obesity, and member of a Data Safety Monitoring Board for a separate Rhythm‐sponsored study of treatment of patients with syndromic obesity. PJW reports a pending patent for metabolic biomarkers of NAFLD/NASH and related disease phenotypes and a pending patent for compositions and methods for treating NAFLD/NASH and related disease phenotypes.

## AUTHOR CONTRIBUTIONS

CJ, JB, MM and OI performed metabolomics and biochemical analysis and interpretation of the metabolomics data. CJ was responsible for writing the manuscript. MB performed advanced statistical analysis; TO and SG consulted on advanced statistical analysis. SA contributed to data collection and database generation. PJW, TO and SG contributed to interpretation of the data and critically reviewed the manuscript. MF was responsible for development of the research question, conception and design of the research project, interpretation of the data and writing of the manuscript. PGB was responsible for development of the research question, conception and design of the research project, obtaining funding, acquisition of data, statistical analysis and interpretation of the data, and writing the manuscript.

## ETHICAL APPROVAL

The Institutional Review Board at Duke University approved the research protocol.

## Supporting information

Table S1

## Data Availability

The data that support the findings of this study are available from the corresponding author upon reasonable request.
