# Supplementary material for: Impact of lifestyle Intervention on branched‐chain amino acid catabolism and insulin sensitivity in adolescents with obesity
Source: Endocrinol Diabetes Metab. 2021 Apr 1;4(3):e00250. doi: 10.1002/edm2.250 (PMC8279626; doi:10.1002/edm2.250)
Supplement: Supplementary file 1 — Table S1 [file EDM2-4-e00250-s001.docx]

| **Supplemental Table 1.** **Comparisons of Baseline Anthropometric Values and Metabolites among participants with and without follow up** | | | |
| --- | --- | --- | --- |
|  | Subjects w/o follow up Mean (SE)  n=49 | Subjects w follow up Mean (SE)  n=33 | P value |
| **Anthropometric Values** |  |  |  |
| Age, years | 14.47 (0.23) | 14.20 (0.25) | 0.4328 |
| BMI | 35.65 (0.86) | 34.67 (1.17) | 0.4930 |
| BMI % | 98.59 (0.22) | 98.31 (0.29) | 0.4416 |
| BMI Z-score | 2.33 (0.05) | 2.27 (0.07) | 0.4530 |
| BMI% exceeding the 95^th^ percentile | 132.25 (2.96) | 129.75 (4.14) | 0.6149 |
| % BF | 41.71 (1.03) | 41.21 (1.49) | 0.7738 |
| Systolic BP, mmHg | 118.26 (1.93) | 120.30 (2.08) | 0.4736 |
| Diastolic BP, mmHg | 63.55 (1.27) | 64.73 (1.66) | 0.5676 |
| **Insulin Sensitivity Measures** |  |  |  |
| Adiponectin, μg/mL | 13.92 (0.72) | 15.12 (0.94) | 0.3100 |
| HOMA-IR | 3.90 (0.30) | 3.70 (0.52) | 0.7387 |
| TG to HDL ratio | 2.14 (0.23) | 1.85 (0.22) | 0.3752 |
| **Metabolites** |  |  |  |
| Glutamate/glutamine μM | 82.59 (2.41) | 81.31 (3.62) | 0.7587 |
| Valine, μM | 278.84 (6.21) | 267.39 (7.94) | 0.2549 |
| Leucine/Isoleucine, μM | 191.75 (3.63) | 186.75 (5.21) | 0.4182 |
| BCAA, μM | 470.60 (9.34) | 454.14 (12.41) | 0.2844 |
| C2 acylcarnitine, μM | 6.18 (0.33) | 6.43 (0.50) | 0.6740 |
| C3 acylcarnitine, μM | 0.37 (0.02) | 0.34 (0.02) | 0.4138 |
| C5 acylcarnitine, μM | 0.12 (0.01) | 0.12 (0.01) | 0.6048 |
| C2 to (C3 + C5) acylcarnitine μM | 13.61 (0.87) | 14.98 (1.28) | 0.3607 |
|  |  |  |  |
